# “Hi This Is NESTORE, Your Personal Assistant”: Design of an Integrated IoT System for a Personalized Coach for Healthy Aging

**DOI:** 10.3389/fdgth.2020.545949

**Published:** 2020-10-14

**Authors:** Filippo Palumbo, Antonino Crivello, Francesco Furfari, Michele Girolami, Alfonso Mastropietro, Giorgio Manferdelli, Christina Röcke, Sabrina Guye, Antoni Salvá Casanovas, Maurizio Caon, Francesco Carrino, Omar Abou Khaled, Elena Mugellini, Enrico Denna, Marco Mauri, David Ward, Paula Subías-Beltrán, Silvia Orte, Ciprian Candea, Gabriela Candea, Giovanna Rizzo

**Affiliations:** ^1^Institute of Information Science and Technologies of the National Research Council of Italy (ISTI-CNR), Pisa, Italy; ^2^Institute of Biomedical Technologies of the National Research Council of Italy (ITB-CNR), Segrate, Italy; ^3^University Research Priority Program “Dynamics of Healthy Aging” of the University of Zurich, Zurich, Switzerland; ^4^Fundació Salut i Envelliment, Universitat Autònoma de Barcelona, Barcelona, Spain; ^5^University of Applied Sciences and Arts of Western Switzerland HES-SO, Fribourg, Switzerland; ^6^FLEXTRONICS DESIGN SRL, Milan, Italy; ^7^eHealth Unit, Eurecat, Center Tecnològic de Catalunya, Barcelona, Spain; ^8^ROPARDO SRL, Sibiu, Romania

**Keywords:** e-health, virtual coach, IoT, sensor network, digital health

## Abstract

In the context of the fourth revolution in healthcare technologies, leveraging monitoring and personalization across different domains becomes a key factor for providing useful services to maintain and promote well-being. This is even more crucial for older people, with aging being a complex multi-dimensional and multi-factorial process which can lead to frailty. The NESTORE project was recently funded by the EU Commission with the aim of supporting healthy older people to sustain their well-being and capacity to live independently. It is based on a multi-dimensional model of the healthy aging process that covers physical activity, nutrition, cognition, and social activity. NESTORE is based on the paradigm of the human-in-the-loop cyber-physical system that, exploiting the availability of Internet of Things technologies combined with analytics in the cloud, provides a virtual coaching system to support healthy aging. This work describes the design of the NESTORE methodology and its IoT architecture. We first model the end-user under several domains, then we present the NESTORE system that, analyzing relevant key-markers, provides coaching activities and personalized feedback to the user. Finally, we describe the validation strategy to assess the effectiveness of NESTORE as a coaching platform for healthy aging.

## 1. Introduction

We live longer and healthier than ever before. This trend is confirmed year after year by the proportion of people aged over 65 populating our countries. The prolongation of the life expectancy is also followed by an increase of the expected quality of the life (1). People aim at remaining active, healthy and autonomous as much as possible. Such expectations, however, represent challenges hard to meet. In this regard, the e-Health systems have the main responsibility to ease such transition, also referred to as *healthy aging*.

However, healthy aging involves several domains. Physical, mental, emotional, and cognitive are all areas that need to be considered during the design of ICT-based solutions for older adults. Most of the commercial solutions available on the market are designed to address only a subset of such domains. This is the case of apps for tracking the sport activities or for improving the mental skills. In such cases, users are required and motivated to increase only some skills to our knowledge, only one solution tackles the healthy aging as an holistic process in which all the domains have to be properly *trained* day by day. This example is provided by the Matilda system (2, 3) based on a robot for older adult care in residential facilities. It proposes different coaching activities for each domain but it doesn't rely on a multi-domain coaching model and the proposed activity are not based on international guidelines. This work describes NESTORE, a companion for older adults, whose main goal is to collect and analyze data from the end-users and to return to them personalized coaching and feedback. NESTORE is a European project, funded under the Horizon 2020 programme, based on a consortium of 16 partners^1^.

The innovative idea of NESTORE is to face healthy aging by simultaneously taking into account four key domains of well-being, shaping the human life in terms of both status and behavior: physiological status and physical activity behavior, nutrition, cognitive and mental status, social behavior. To deal with the complexity of aging changes, NESTORE coaching focuses on one specific aspect for each well-being domain: physical activity, nutrition, cognition, and social relations. In this work, we describe our vision of personalized coaching with a set of comprehensive application scenarios as well as the design of the NESTORE IoT-based architecture used to coach the end-users. Our solution firmly relies on unobtrusive sensing devices able to collect data from different domains, for example, tracking physical activity, detecting nutrition elements, assessing sleep quality (4) as part of the physical domain or recognizing social interactions with a fine-grained temporal resolution. In turn, such information is analyzed by a Decision Support System (DSS) able to deliver, through the Virtual Coach, personalized feedback to the NESTORE users. The design of NESTORE follows a structured methodology. First, we define a physiological, behavioral, and psychological model of the prospective older adult user through the involvement of domain experts. Second, we identify those markers to be monitored through IoT-based devices. In this vein, it is worth noticing the design of a custom hardware for detecting some physiological markers as well as the use of commercial devices. Finally, we evaluate the key health and well-being markers both on the short- and long-term period in order to provide the final users with specific coaching plans. The NESTORE approach is under evaluation in 3 pilot sites across Europe: Spain, The Netherlands, and Italy. Each site involves 30 (20 in the experimental and 10 in a control condition) individuals recruited on a voluntary basis and followed by experts during the NESTORE coaching intervention.

The rest of this work is organized as follows. Section 2 shows the background of NESTORE with related work and initiatives. Section 3 explains the core coaching mechanism and its areas of intervention. Section 4 details the software and hardware infrastructure supporting the overall system. The validation of NESTORE is detailed in section 5, while section 6 draws the conclusions.

## 2. Background and Related Work

In the last decade, a growing number of research projects and commercial products have dealt with the development of e-Health solutions to promote and to support well-being with a focus on the so called “silver economy” scenario (5).

Several EU funded projects have worked on ICT solutions to early detect risks in different aspects of older people's lives. For example, the main ambitions of the EU H2020 My-AHA project^2^ are the early risk detection and intervention in order to support healthy aging both in the physical and cognitive domain. The project relies on the deployment of Ambient Assisted Living (AAL) sensors, wearable devices, and smartphones, and through big data analysis is able to engage users in improving their lifestyle both in a short-term and long-term vision. In the same context, EU H2020 projects GrowMeUp^3^ and Radi^4^ provide integrated and services through robotic-based approaches. These two projects mainly monitor the behavioral trend of a healthy older population. The main goal is to support and to encourage older persons to stay active longer. PreventIT^5^ and REACH^6^ are focused on monitoring the users' physical activity through the deployment of wearable and/or ambient sensors. Additional European research projects on the coaching of the elderly which feature common elements to NESTORE are: The CAPTAIN System (6) providing a projected and tangible interface; COACH Council of Coaches (7) with multiple autonomous, embodied virtual coaches; HOLOBALANCE (8) with their new personalized hologram coach platform.

The NESTORE project is intended to overcome the limitations of vertical solutions (single-domain oriented works) with a strong holistic approach by addressing several singular domains (cognitive, physiological, social, and nutritional) (9–11) and by considering these domains from a user-centered approach. Furthermore, in order to support healthy aging, the NESTORE ecosystem provides a tangible interface to improve human-machine interactions and the acceptability of the proposed solution.

From the commercial point of view, several products have been presented in the last years to hit the market of e-Health coaching systems. In general, these solutions require a human-machine interaction through tangible objects enriched with artificial intelligence capabilities or through smartphone applications. The most important interface that a tangible object presents is based on voice interaction (e.g., Amazon Alexa, Google Speech, IBM Watson Conversation, Microsoft Bot Framework). The voice interaction is generally well-accepted by older adults and increases the accessibility of the interfaces for people who start to have perceptual impairments (12). On the other hand, smartphone-based applications offer advice and coaching activities through messages, notification, reminders, and tips. For example, the growing market of coaching systems for athletes (e.g., runners, bikers) contains several applications able to give personalized suggestions to the user in order to improve their performance or to set up a training schedule (13). The main limitation of these approaches is that they are usually focused on a single domain. However, addressing human well-being and promoting the independence of the older population is inherently a multi-domain challenge.

### 2.1. Requirements for an E-Coaching Intervention

With respect to traditional e-Health interventions, a system that monitors user's behavior and provides personalized suggestions to improve health-related outcomes can be called virtual coach. Such a coach can be simply embedded in smartphone devices (e.g., app), but can also have a more anthropomorphic and physical embodiment, for example in form of an Embodied Conversational Agent (ECA) or a robot. As defined in the introduction to a recent IEEE Computer special issue on E-coaching for Health (14), coaching a user means to “frequently, but not continuously, observe, listen to, question, understand, reason with, teach, and/or advise the users in order to change their behavior and to improve their health.” To this purpose, they continue, “intelligent systems are used to encourage progress toward specific health-related goals by providing tailored training and guidance.” While in (15) are suggested 9 important features that an e-coach system should have (i.e., social ability, credibility, context-awareness, learning abilities, data gathering, proactivity, reflection, behavior change model integration, planning support), authors in (16) reviewed the key components that can significantly affect a variety of health outcomes, the adherence and the usability of an e-coaching intervention. The following behavior change techniques were found to positively affect both health outcomes and usability in the studies reviewed:

- Setting short-term goals to eventually reach long-term goals;
- Personalization of goals;
- Praise messages;
- Reminders to input self-tracking data into the technology;
- Use of validity-tested devices;
- Integration of self-tracking and persuasive e-Coaching;
- Provision of face-to-face instructions during implementation, as key components for influencing both health outcomes and usability in a positive way;
- Provision of personalized content.

## 3. A Personalized Coaching Experience

**Figure 1:**
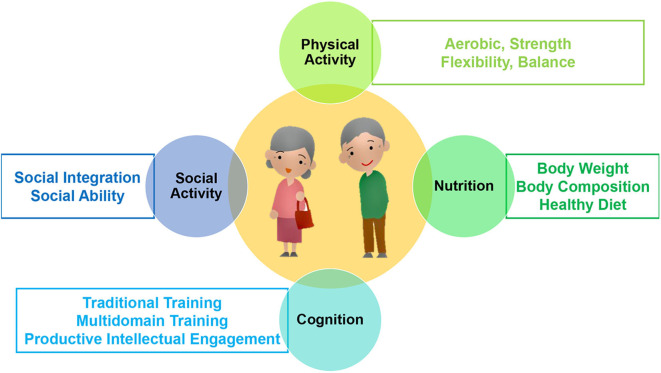
The NESTORE coaching domains and subdomains.

The NESTORE system is mainly aimed at providing multi-domain coaching plans specifically tailored to the users' needs in order to maximize their engagement and health benefits. NESTORE is focused on physical activity, nutrition, cognition, and social relations, which are composed by specific subdomains, in order to consider, as much as possible, the factors that must be monitored and the abilities that must be strengthened during physiological and psychological aging (Figure 1). The NESTORE users, after a preliminary phase of assessment which is intended to characterize the subject's status and behaviors using ICT tools, are invited to select personalized coaching pathways in order to improve or maintain their skills in each specific domain.

Once the assessment is performed, different “pathways of well-being” are set up as “tracks” within users make their journey experiencing the NESTORE coaching system (17). A pathway of well-being is the process of pursuing a high-level goal to which the user will commit at the end of the motivational phase. Since well-being is intrinsically multi-domain, a pathway spans across multiple areas (e.g., I want to maintain my everyday mental skills while improving my physical activity and nutrition). Each domain is quite complex and training every aspects of it could be overwhelming or practically impossible. For this reason, we divided each domain into sub-domains (e.g., the Nutrition domain is divided in “Decrease body weight,” “Achieve a healthy diet,” and “Maintain muscle mass”). Therefore, a pathway could be seen as a selection of sub-domains based on system recommendation (based on the user's status) and user preferences. Once the user chooses a pathway, a set of coaching activities related to the pathway is proposed. A coaching activity is a time-bound activity that can be scheduled in the personal calendar (thus helping the user in planning the activity and sticking to the plan). The activity can be composed of a set of specific exercises (e.g., flexibility exercises), or can be proposed as an everyday activity (e.g., gardening with a friend, or buying vegetables in a supermarket). In both cases, instructions for carrying out the activities and maximizing the impact in the involved domains are provided. Coaching activities are defined by the experts in each of the NESTORE coaching domains. They can be defined as structured activities (i.e., specific exercises carried out to train the user in a specific domain) or non-structured activities (i.e., everyday activities that can be conducted as part of the daily routines but that can still contribute to the improvement of well-being in one or more domains). The unstructured activities are intended to avoid forcing the user on performing unwanted activities; thus they are chosen from a list of actions (dancing, walking with the dog, etc.) that the user likes to perform during his/her everyday life and are suggested by the system based on the specific user's choices, also considering the feasibility of performing the activity itself in a specific place and moment of the day. However, if the user does not meet the prescribed coaching recommendations with the only unstructured activities, the system proposes also structured activities (for example running or walking in the case of aerobic physical activity) to ensure that the required prescription, as defined by the international guidelines, is reached. While pathways will be defined in the system as a list of coaching activities, such list can be customized by the system according to user status, context and preferences. For example, regarding the physical activity, the user can select the weekly frequency of the sessions and the intensity level so that the duration of the daily session is automatically adjusted, in order to meet the needed prescription (18). After the completion of the activity, the user can review his/her personal trajectory in the system. Eventually, the system might have recognized a part or all the training activities performed. The user can still modify what was recognized by the system or add additional information (self-reporting).

**Figure 2:**
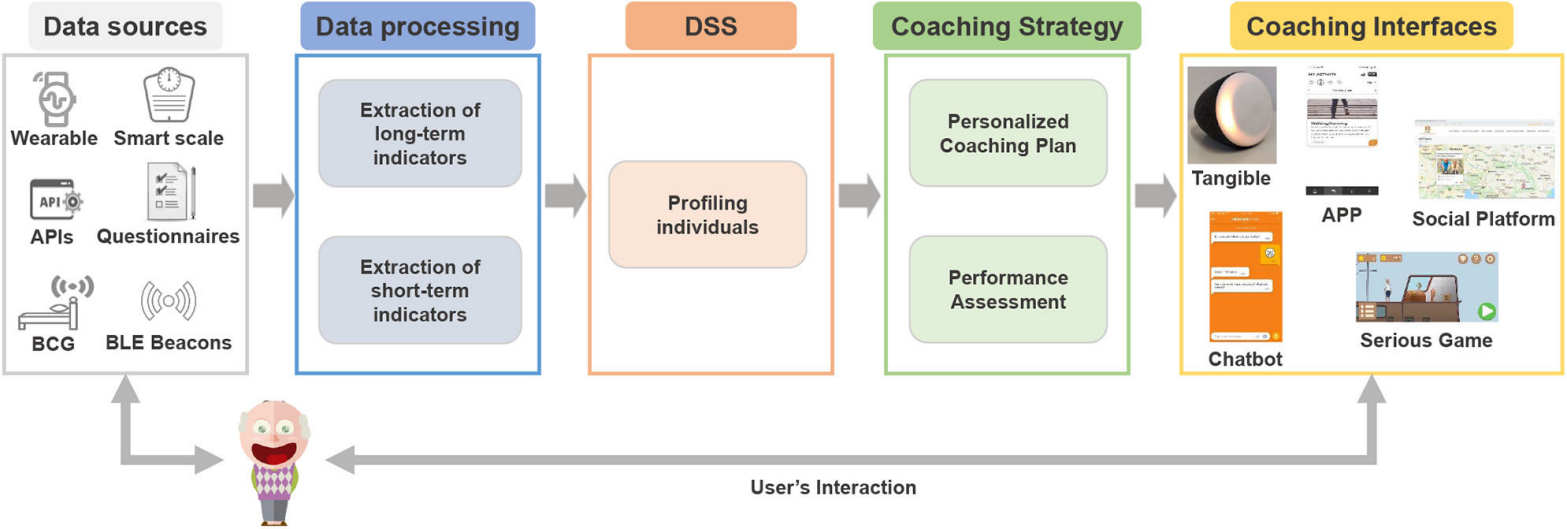
The NESTORE coaching system.

The NESTORE system includes a plethora of sensors and other data sources (wearable devices, environmental sensors, questionnaires, APIs), which measure data directly from the users and process it to extract short-term and long-term indicators. The raw data, as well as the indicators, are then elaborated by a DSS to profile the users using static and dynamic variables. The users' profiling is fundamental to propose personalized coaching plans and performance assessment. Finally, the users interact with the NESTORE coaching interfaces (tangible, chatbot, APPs, serious games, social platform), in order to receive clear instructions and feedback to perform the prescribed activities composing the whole coaching plan correctly. A schematic representation of the NESTORE coaching system is shown in Figure 2.

### 3.1. Physical Activity

The physical activity coaching is focused on four sub-parts: (i) aerobic training, consisting of structured activities, such as walking, running, or cycling to improve cardio-respiratory fitness; (ii) strength exercises, such as chair squats or knee push-up to improve upper and lower limbs strength and power; (iii) flexibility training, using stretching exercise for upper and lower limbs to improve the body range of movement; and (iv) balance exercises, such as one leg stand or toe-to-line to improve the overall body stability. The NESTORE coaching system works by scheduling structured sessions in the user's agenda; the structured activities are individualized according to results obtained by some standard physical performance tests, which are performed in the monitoring phase and every 4 weeks. Two main pathways can be followed according to users' needs of retaining or improving their physiological status. A score system assigned to each training activity will allow keeping track of user progress and personal goal attainment for the selected pathway and in all the involved domains. Such scores can be shown to the user or not, according to his/her preferences.

To be compliant to the suggested coaching plans, the user should reach a total weekly score, by performing structured and/or non-structured activities. The overall weekly score for the domain is 100 points weighting every single sub-part considering its importance for the subject quality of life and execution of daily life activity (30 points for aerobic training; 50 points for strength; 10 points each for both flexibility and balance). Each activity has its own score that is calculated considering the impact of that specific activity in the related well-being domain. For example, 10 min of aerobic activity performed with moderate intensity corresponds to 2 points for aerobic training. Similarly, dancing for 1 min corresponds to 2 points for the aerobic training, sweeping corresponds to 2 points for aerobic training and 0.33 for strength, whereas walking with the dog corresponds to 1 point for aerobic training and 0.33 points for strength. The different components (i.e., structured and non-structured activities) add up to generate the overall score. This implies that the more non-structured activity a user performs the less structured activity is suggested to the user by the system. This choice is intended to make the system less intrusive as possible concerning the daily life usual activities of the users.

In order to assess if the user is performing his/her physical activity at best, the wearable device embedded in NESTORE is able to monitor the intensity of the structured activity during the whole exercise session and to provide a real time feedback (vibration) to the user if the heart rate is too low or too high. Furthermore, at the end of the training session the system determines the adherence of the user to the prescribed plan. Additionally, to avoid an excessively strenuous training, the system, after each session, asks the user to provide the Borg Scale and the Total Quality of Recovery scale as indicators of perceived fatigue and recovery. Based on these user feedback, the system can then adapt the intensity and the duration of the next exercise session in order to maintain the weekly correct prescriptions.

### 3.2. Nutrition

The coaching plans for the nutrition domain are structured as follows: (1) body weight management, focused to increase or decrease body weight, and consisting of tailored dietary activities, as well as energy balance monitoring, (2) body composition management, focused to increase muscle mass or decrease body fat, and consisting as well of tailored dietary activities and energy balance monitoring, and (3) healthy diet, focused to improve their dietary habits by targeting macro-nutrients and micro-nutrients intake through both diet monitoring (based on automated food image recognition) and nutrition coaching. The DSS will suggest one of the aforesaid coaching plans based on the anthropomorphic measures of users (i.e., weight, height, BMI, fat free mass, muscle mass, and waist circumference) and their nutrient intake evaluated at baseline. Once the coaching starts, users will receive recommendations of food to include in their diet, recipes to cook, and good practices to follow taking into account the foods they refuse (e.g., none gluten food will be recommended to celiac people) and their nutritional habits. The DSS will schedule grocery store outings with the ingredients that will be needed in the upcoming days. Those users that track their food intake periodically will receive more customized recommendations that those that do not.

### 3.3. Cognition

In the cognitive domain, the NESTORE coaching consists of three plans, (1) one focusing on memory (involving a structured traditional cognitive training task in the domain of working memory), (2) one focusing on broader thinking skills (involving a multi-domain serious game that involves simultaneous tasks in the areas of visuo-motor ability, spatial memory and inhibition), and (3) one that targets everyday cognitive functioning (involving productive intellectual engagement in the form of unstructured leisure activities that require learning a novel and complex skill, such as digital photography, dancing, etc.). The DSS will recommend coaching in the cognitive domain based on pre-defined performance thresholds in the cognitive tasks assessed at baseline. Those users whose cognitive performance is above the critical threshold will be given the recommendation to keep up their current lifestyle concerning activities as is so as to maintain their cognitive functioning. Those individuals below a critical threshold will receive the recommendation to engage in either one of the three cognitive pathways. Performance thresholds are defined based on the typical thresholds used in adaptive testing in cognitive training research, where an accuracy below .6 leads to a decrease in task difficulty and an accuracy above 0.8 leads to an increase in task difficulty. Accordingly, we have chosen to not provide a recommendation for additional training if individuals score beyond 0.8, to provide a first degree of recommendation if between 0.8 and 0.6 and a strong recommendation for individuals below 0.6 accuracy. The tests used at baseline are state-of-the-art psychometric cognitive tests intended for the assessment of cognitive capacity in the domain of working memory: N-back test and numerical updating) in addition to a validated questionnaire on cognitive failures for older individuals with no known diagnosis of cognitive impairment.

### 3.4. Social Activity

In the social domain, the NESTORE system will target two areas: (1) The first focuses on (the improvement of) social integration by suggesting users to join group-based activities in their community in a domain that matches their personal interests (e.g., sports, culture, education, community service). (2) The second pathway targets social skills and their improvement, such as a wide range of communication techniques (face-to-face, computer skills in order to better use email or video calls, tolerance of silence in conversations, giving compliments). This pathway requires to join structured classes in the user's community and is currently not something that is offered as a class within the NESTORE system. Given that through a person joining a new group-based activity communication skills are also trained, the NESTORE system will primarily focus on the first social pathway. Recommendations for the social domain in general will be given by the DSS based on social integration and loneliness screening measures assessed at baseline. Those participants who are well-integrated and not lonely will not receive a particular recommendation in the social domain other than to keep as socially engaged as they currently are. Two standard questionnaires to assess social integration and loneliness with known thresholds are employed at baseline, the De Jong Gierveld Loneliness Scale (19) and the Lubben Social Network Scale (20). Both scales have published thresholds for the identification of at risk (for loneliness) individuals and are widely used in gerontological research.

## 4. The Integrated Infrastructure

**Figure 3:**
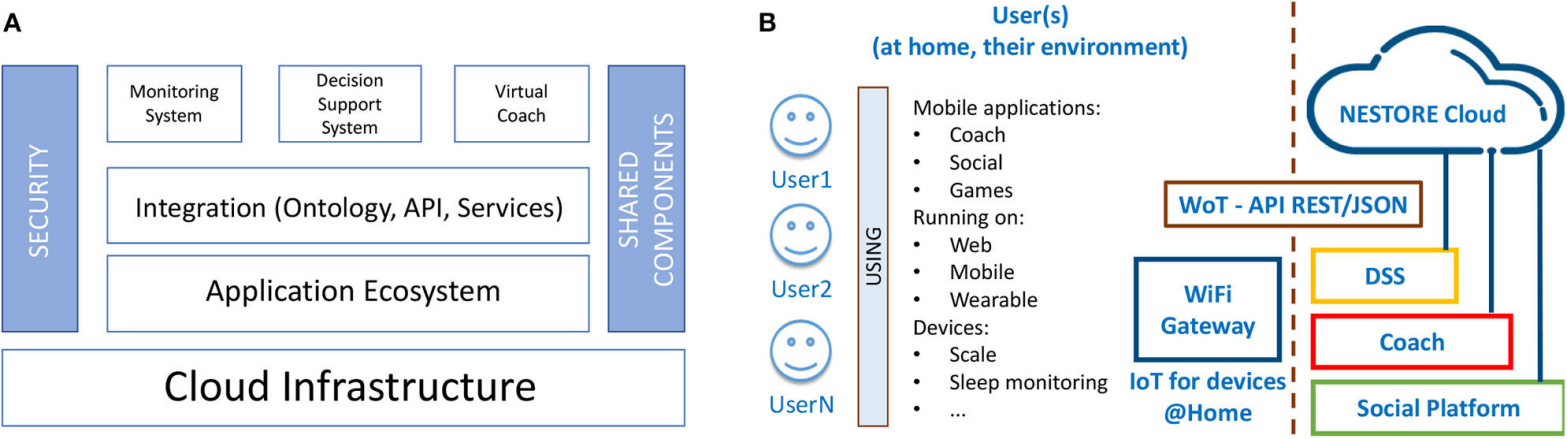
Overall view of the NESTORE integrated IoT architecture: **(A)** shared functional modules and **(B)** used technological components.

The NESTORE project relies on a complex IoT architecture whose goal is twofold: to retrieve sensing information from users and to analyze the data collected so that valuable feedback can be given to users. This section describes the NESTORE IoT architecture that integrates three interconnected and complementary components essential for the e-Coaching activity: the Monitoring System, the DSS and the Virtual Coach (Figure 3A). These components provide a robust solution for easily adding and integrating 3rd party applications.

Shared modules (components) are referred as: Identity Manager, Security Manager, User Profile, and Log Service that are used by the platform and its components. The Identity Manager Module is necessary for end user identities management across the platform components, APIs, the cloud, mobile, and sensors, regardless of the standards on which they are based. On the other hand, the platform exposes a wide range of data, which are subject to security and privacy concerns like: user data, sensor data, actuator access, service membership. Each of these areas must offer a proper security level which is audited for compliance with existing rules. As shown in Figure 3B, the main components of the NESTORE ecosystem are identified, starting with: (a) End Users, they will interact with the ecosystem through their smartphones and by using web applications; (b) NESTORE cloud, it hosts all the back-end parts and oversees retrieving, elaborating and storing the data coming from the platform users and other third-party applications; (c) The End Users are using the NESTORE applications on their environment/their house by accessing specific coaching applications (in form of Web, smartphone or wearable applications) that are running on different devices (tables, phones, wearable) accessing Internet; (d) The environmental sensors are connected to the NESTORE platform using WiFi/3G connection, using the IoT/WoT (Web of Things) approach.

The underlying integrated software infrastructure provides the basis for building the NESTORE system as a whole. From a technological point of view, it can be seen as an ensemble of software and hardware modules that address the functional requirements of the different domains of the coaching experience. The details of the particular non-core solutions, such as sleep monitoring (21), social activity (22, 23), indoor behavior analysis and environmental monitoring (24), can be found in the referenced works, while the core functionalities of NESTORE will be described in the following subsections.

Data are initially collected from user's personal space with a sensor kit following the IoT paradigm (section 4.1). Each kit comprises commercial and custom devices able to sense the environment and to send the data to the back-end through a mobile gateway (section 4.2). The raw data collected are then elaborated on the cloud so that to extract data useful for the DSS (section 4.3). The DSS has the main role to feed the Virtual Coach which implements the interface with the end-users (section 4.4).

### 4.1. The IoT Architecture

An IoT solution is characterized by the connection of several devices (i.e., things) which can use a gateway to communicate on a network to back-end servers running the IoT platform to integrate the information. The roles of the devices, gateways and cloud platforms are well-defined; each provides specific features and functionalities needed for creating a robust IoT solution. The NESTORE architecture supports different types of IoT devices that can connect and push data into the cloud, from Sensors to Applications as defined in (25). Security methods are implemented from the devices to the cloud. Features such as authentication, encryption, and authorization are part of the solution stack.

**Figure 4:**
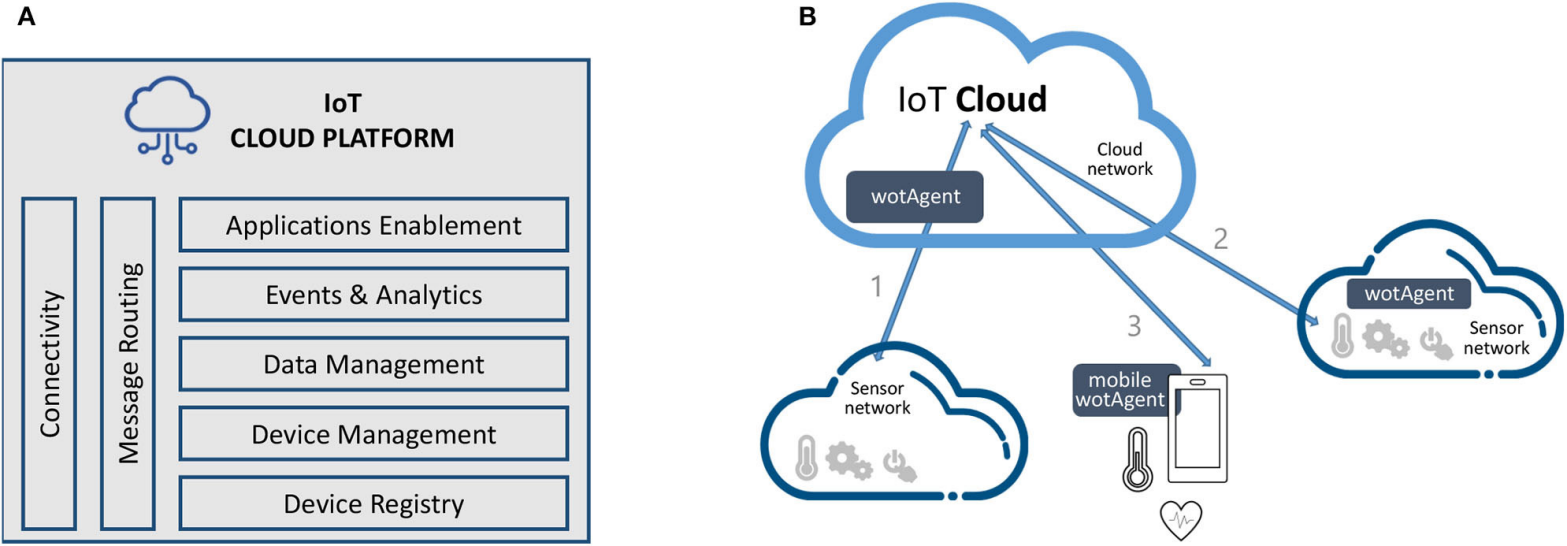
The NESTORE IoT architecture: **(A)** server-side components and **(B)** the Web of Things model.

A high level overview of the proposed architecture is depicted in Figure 4. The device registry component ensures the definition of the device categories and the data type that are supported by each one. In order to assure data analytics and data interoperability for the device data, the format and description of data must be taken into account from the beginning. Thus, a definition of the ontologies and metadata across heterogeneous domains is a key requirement. Device management is responsible for the enrollment of each “thing” with the system and managing each device instance. After a successful enrollment a “thing” can communicate (send/receive) data with the IoT system. The data management module handles data about how the user interacts with the “thing” and it traces its usage among different users. It is assuring data security by separation of data generated by the same “thing” for different users on different time intervals. To take full advantage of the IoT system, the event and analytics module ensures the possibility to define, manage, and use events and basic analytics by integration of the well-known Grafana engine^7^.

Connectivity and message routing are implementing the needed protocols for the communication with the IoT devices. AMQP (Advanced Message Queuing) protocol (26) is used, being a protocol widely used for the type of middleware applications, that implements routing rules, stores messages, and sets their distribution rules, allowing exchange messaging between clients and the server. In the NESTORE context, we identified that data must be transferred to the cloud for the following scenarios (27): (a) Sensor to Device using different protocols and transmission/transport methods involving the Open Systems Interconnection (OSI) level 1 to 4; (b) Device to Cloud and Sensors to Cloud using HTTP/Web Socket approach that implement the OSI level 7 Application layer. A special device is represented by the gateway that enables machine-to-machine communication. All data moving to the cloud, or vice-versa, go through the gateway, which can be either a dedicated hardware appliance or software program. The gateway provides a place to pre-process data locally at the edge before sending it on to the cloud. When data is aggregated, summarized, and tactically analyzed at the edge, it minimizes the volume of data that needs to be forwarded on to the cloud, which can have a big impact on response times and network transmission costs. REST web services are used to access the IoT system. The proposed architecture defines and implements a common strategy Application Programming Interface (API) for managing the IoT platform and for acquiring data from various devices. Through APIs, it is possible to send data from devices (e.g., sensors, mobile apps, etc.) to the cloud by Web of Things devices or gateways. Reusing and adapting patterns commonly used for the Web, we enabled the IoT architecture as Web of Things, using Web servers (28) on smart things and applying the REST architectural style (29, 30) to the physical world. The essence of REST is to focus on creating loosely coupled services on the Web so that they can be easily reused (31).

A device to be connected to the IoT platform needs to implement a Web of Things Agent (wotAgent) that transforms a sensor or a device into a compatible WoT element. The wotAgent can be configured at the Cloud level when the wotAgent support OSI level 3 and 4 and it translate the data to the cloud from the sensors and devices. When it is configured to run on a mobile device on top of an operation system (i.e., Android) or transforming a device/sensor into a smart device then wotAgent implements locally the OSI level 1 to 4 and it communicate with the IoT cloud using REST. Figure 4B shows the wotAgent model adopted in NESTORE.

### 4.2. The Monitoring System

The kernel of the NESTORE system is developed around a wearable and environmental devices. The wearable device and its charging station are part of the multi-sensor monitoring system of NESTORE representing a multi-parameter observatory and data recorder in the form of a wristband together with Bluetooth Low Energy (BLE) beacons. The devices communicate via a BLE chip with both wearable and environmental beacons as well as smartphones and tablets. The wristband can work for about 18 consecutive hours considering a standard use case expected by Nestore pilot. Battery level status is always visualized qualitatively with an icon on the wristband screen and the low battery level status is signaled with a specific alert notification and a buzz. Charging station takes about 5 h to completely recharge the wristband battery and during the recharging process an animation is showed on the wristband display to inform the user about the charging progress.

**Figure 5:**
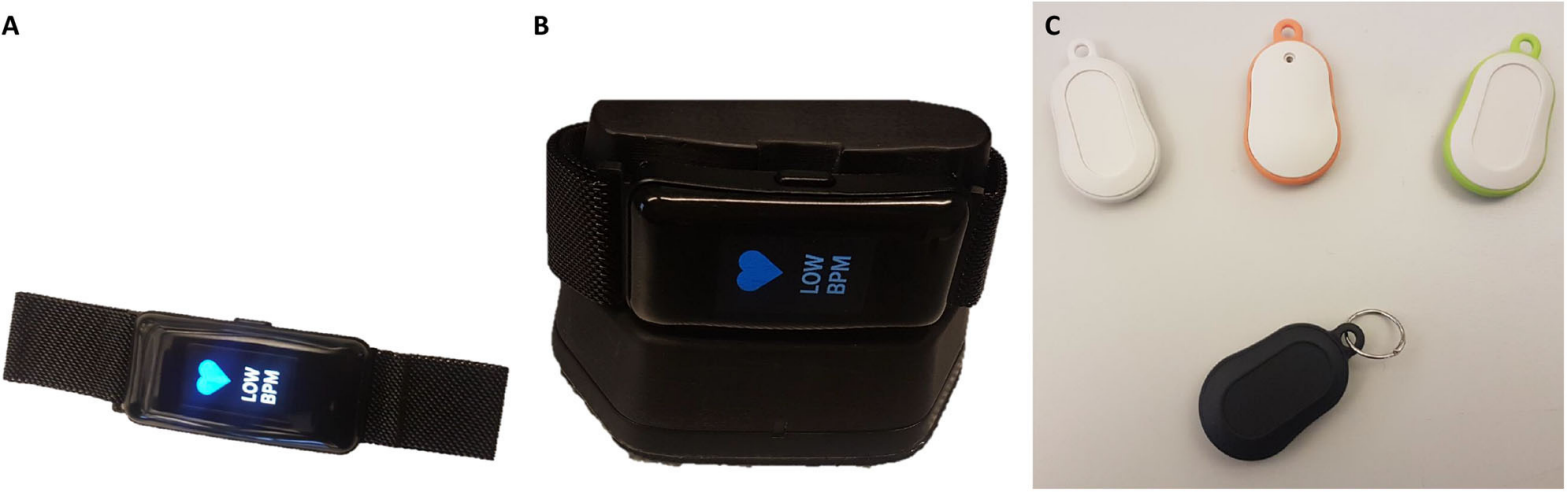
The NESTORE monitoring artifacts: **(A)** the wearable device; **(B)** the charging station; **(C)** the social and environmental beacons.

BLE beacons are deployed in the user's environment to detect social interactions among NESTORE users and their relatives (bringing with them keyfobs equipped with mobile BLE tags) with their duration, function, location, and number. We exploit the capability of calculating the proximity between BLE devices from Received Signal Strenght Indicators (RSSIs) (32–34) also for detecting the interaction of the user with pieces of furniture in the house. We deploy fixed beacons that give us insights on the users level of sedentariness (35, 36). We customized the NESTORE BLE beacons deployed in the house to advertise additional information like motion (to increase the level of accuracy in detecting interactions with point of interests in the house), temperature and humidity [to calculate the indoor air quality indicator (37)]. In this context, the wristband also acts as a hub for the beacons (*W* for wearable beacon and *E* for environmental beacon). Function and features for the wristband and beacons vary and are summarized in Table 1. The wearable device is recharged with a dedicated wired battery charger while the beacons have replaceable coin-cell type batteries as shown Figure 5.

**Table 1:** Functions and features of wristband/beacons.

**Function and feature**	**Wristband**	**Beacon**
User steps counter	✓	
Distance estimation	✓	
Activity type recognition (i.e., “no activity,” walking or running)	✓	
Sedentariness monitoring	✓	
Stairs (Up and Down)	✓	
Energy expenditure	✓	
RSSI		W and E
Battery level monitoring		W and E
Motion detection		E only
Temperature monitoring		E only
Humidity monitoring		E only

As with all system engineering driven projects, the starting point is the definition of the user, user journey, usage environment and stakeholder needs and relevant requirements (38). Since NESTORE is a human-engineered system, the first batch of requirements concerns the overall business mission of the system described in the business requirements specification (BRS). This is followed by stakeholder requirements (StRS) and concludes with systems (SRS) and relevant sub-system requirements. The BRS represent the business intention of NESTORE (denoted “enterprise”) that connects the external environment or market needs or the Concept of Operations (ConOps) while the subsequent StRS and SRS represent the operations concept (OpsCon) (39). The opening high-level project business, stakeholder and system requirements specifications are simplified and summarized in Table 2.

In deriving the user perspectives concerning:

- Identification of needs, values and suggestions for co-design;
- Transferability of participants' perspectives to technologists;
- Co-design user-end participation for prototype improvement;

**Table 2:** Business (BRS), Stakeholder (StRS), and System Requirements Specifications (SRS).

**BRS**	**StRS**	**SRS**
Business model of NESTORE	Wearable bracelet	Wristband status-diagnostics:
		1. Sensor status
		2. Charging status
		3. Battery level status
Development challenges	Easy-to-use and small wearable device	Wristband alerts:
		4. Low battery
		5. User sedentariness
		6. Wristband “not worn”
		7. Connection needed
Development time and cost	Non-invasive	Wristband data availability:
		8. Non-structured activities
		9. Sedentariness periods
		10. Social interactions
		11. Environmental interactions
		12. Structured activities
		13. Evaluation tests
		14. Device error logs
Type and number of users	Does not complicate user everyday life	User characteristics:
		15. Age
		16. Gender
		17. Weight
		18. Height
		19. Resting heart rate
		20. Maximum heart rate
		21. Language preference, etc.
Innovation brief	Mobile app provision	22. Battery duration
User experience-user journey	Ease of maintenance/installation	23. Battery recharging time
User needs and user definition	Data security	24. Display size and type

these were provided by the relevant co-design specialists involved in the project and then translated into an overall Industrial Design (ID) framework. This led to the generation of three wristband concepts and a charging station as shown in Figure 6.

**Figure 6:**
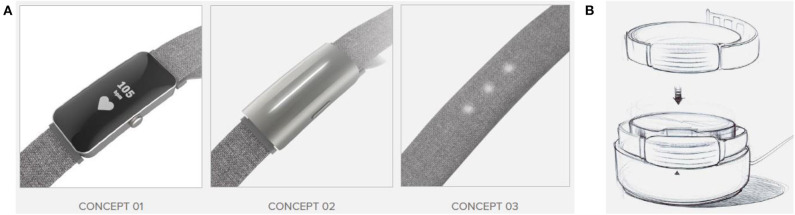
Graphical ID framework translation of user input from co-design specialists report: **(A)** wearable device and **(B)** charging station.

**Figure 7:**
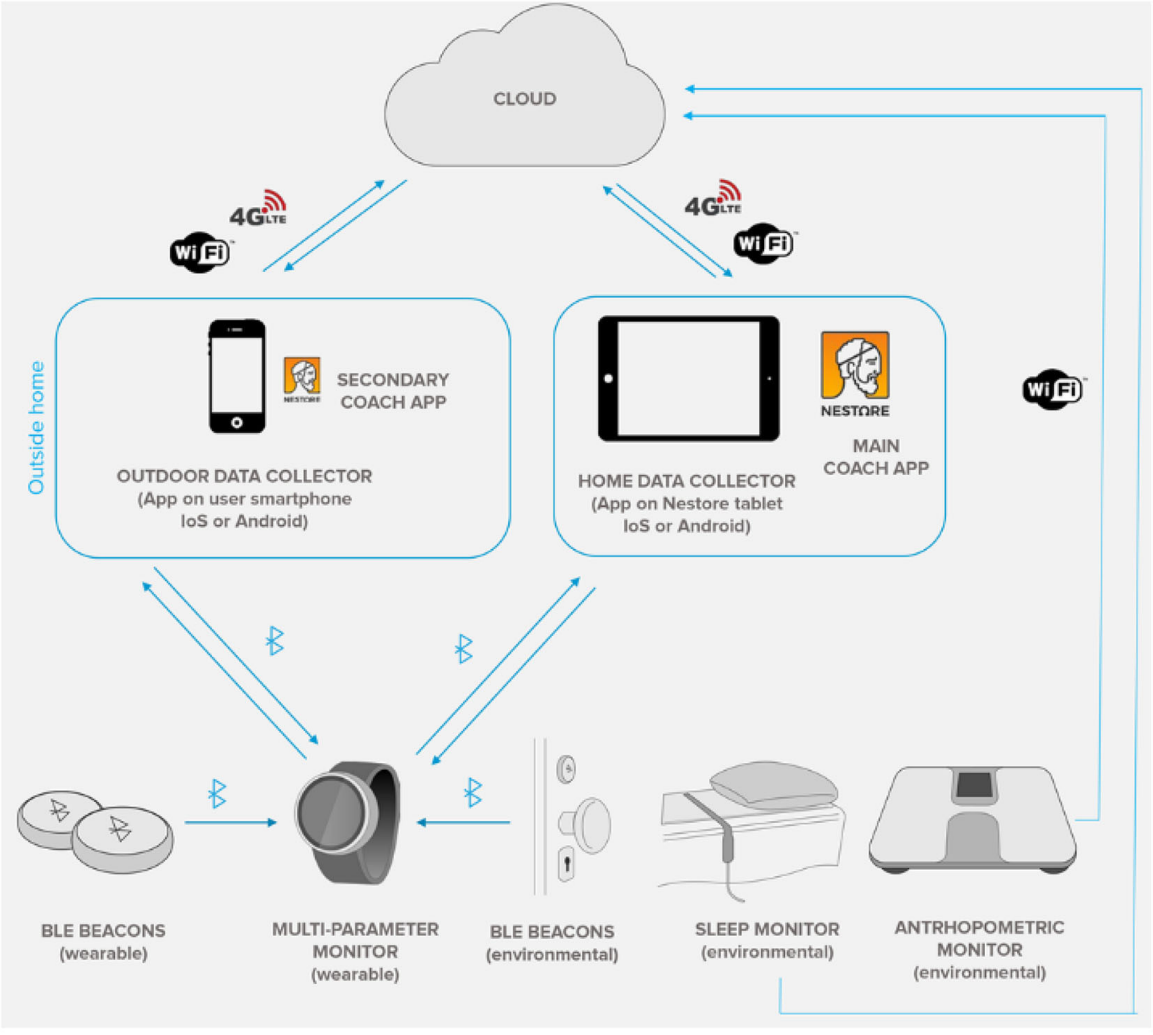
Overview of the monitoring system architecture.

To complete the NESTORE monitoring system there is also a smart scale to collect anthropometric, musculoskeletal characteristics, and balance (40) and a ballistocardiographic (BCG) system in order to perform sleep monitoring (41–43). The NESTORE system and the outline of the device interactions is illustrated in Figure 7.

### 4.3. The Decision Support System

The DSS can be considered the intelligence of NESTORE (18). Its aim is to provide tailored coaching to users based on integrated data sources encompassing the four target domains. Once this data is processed, it is inputted into the DSS so a customized coaching intervention can be designed for each user. The DSS is comprised by multiple modules:

- *Profiling individuals*: A complete profile of users is built based on the extracted indicators, which include demographic and environmental data, information about their preferences and habits, and data describing their nutritional, social, physical, and cognitive status and behavior. A user's profile is translated into tags that capture the information that will be used to personalize the given coaching.
- *Pathway suggestion*: Rule-based Reasoning (RBR) is used to assess users' status in relation to all pathways. The standardization of the thresholds between pathways comes with a three-level scale to measure the users' status per pathway.
- *Coaching and the tagging system*: After users choose the pathways to focus on, the tagging system comes into play by proposing suitable Coaching Events (CEs) to users.

These three modules use AI techniques covering a wide range of methodologies: regression analysis to infer the daily routines of users; statistical modeling to measure the similarity among users; RBR to select the most adequate pathway to recommend (44).

Personalization in NESTORE has the following dimensions: tags to describe users and CEs, ratings, and history analysis. Tagging is the process of assigning meta-data to content in the form of keywords. Users' profiles are tagged automatically thanks to a given ontology and expert driven-criteria. Tags together with ratings are inputted into the tagging system, which employs recommendation techniques to tailor coaching events to users. History analysis is meant to assure diversity in the recommendations given.

**Figure 8:**
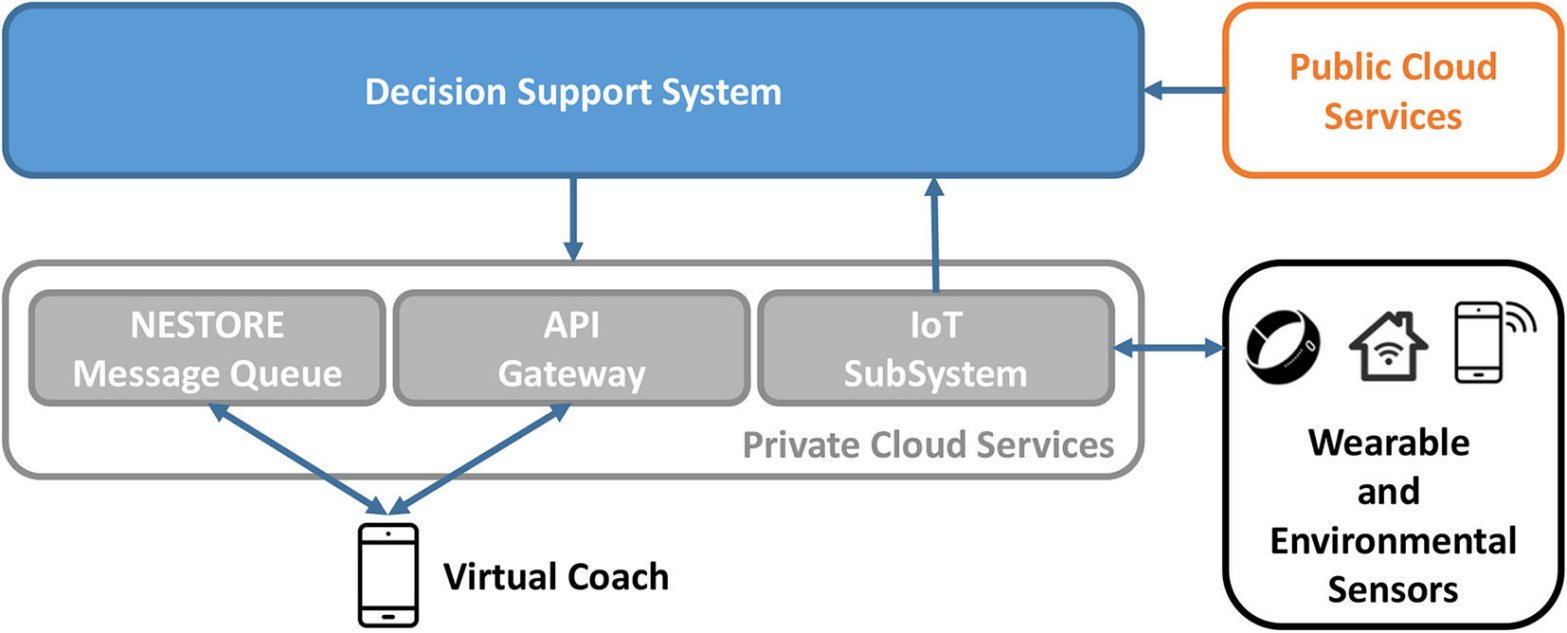
Overview of The DSS architecture.

The analysis provides a further level of understanding of the status of the final users, which helps in personalizing all the interaction with the coach. As depicted in Figure 8, the DSS is fed by different Public Cloud Services as the LogMeal API and the information coming from the wearable devices and sensing units through the IOT SubSystem. They can be grouped as shown next:

- Sensing system: It is composed of a wearable device and an ensemble of environmental wireless devices that detect the status of the users' living space and their behavior. Those include a smart scale, a BCG for sleep quality, and BLE beacons.
- Food recognition: The LogMeal API is used to automatically build a food diary based on images captured by the users.
- Questionnaires and tests: A psychological model is built based on the social and cognitive profile of users. These forms are also used to describe users' preferences and likings.
- Serious games: Cognitive and physical exercises are suggested through game-based initiatives to foster well-being in an entertaining way.
- Context: Personalization is improved by adapting the coaching events (CEs) to users' daily life contexts, e.g., by using meteorological or location-related data.

### 4.4. The Virtual Coach

The NESTORE project aims at presenting a new concept of an e-coach, which consists of an artificial intelligence that is able to monitor the user's behavior through an extensive and heterogeneous network of sensors and devices, and that can produce an intervention aimed at improving the user's behavior considering all the peculiar characteristics of that specific individual. The NESTORE e-coach is able to communicate with the user via multiple multimodal interfaces, dynamically adapting the interaction to the type of content, the contextual situation and the user's current activity. The e-coach interacts with the user using different modalities in a context-aware manner. For example, Jana, who is 68 years old and retired wants to increase her social integration. The e-coach then proposes a list of fitting activities via the chatbot, where she can ask about all the details of the proposed activity list. Moreover, the mobile app allows to create an event choosing the preferred parameters, directly sharing with her network and creating an appointment in her calendar. Once the invitations to her social network are sent, Jana can go back to her daily routine and for this reason she activates the tangible interface with a gesture. While cooking, she can casually chat with the tangible interface. The vocal interaction is quite convenient while the user is occupied in other tasks that require her attention. Jana can ask the e-coach through the tangible interface if any of her friends have already accepted her invitation. Once she will be done eating, she can prepare to go to bed and with a simple gesture she can put the tangible interface in sleep mode.

**Figure 9:**
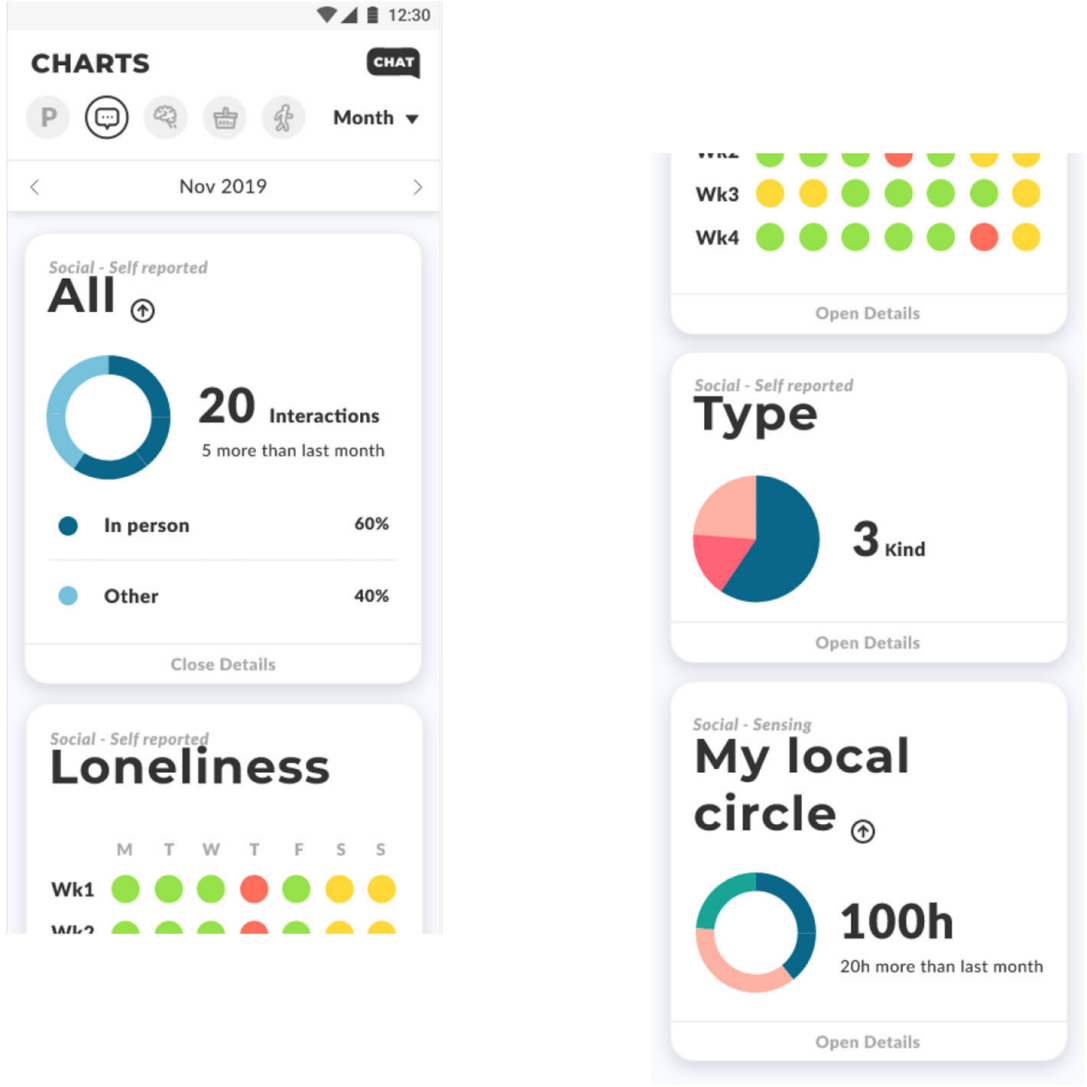
Feedback on the Social domain via the app interface.

Within NESTORE's mobile app, the interaction with the user is mainly managed by five interconnected interfaces: the application, the social platform, the chatbot, the serious game, and the tangible coach. The mobile application represents the main entry point for the user to interact with the NESTORE system. Its main role is to provide feedback on the different domains of the current pathway and to help schedule the activities. Once logged in, the app presents a quick overview on the progressions made in the current pathway. The user can then navigate to get a more detailed view of the achievement and stats in all four domains. Each of these views has a specialized interface developed with the support of the domain experts. These views show several dimensions of the user's temporal evolution (daily, weekly, and/or monthly). For instance, the social view (Figure 9) shows stats about perceived loneliness, the type of social interaction having taken place (by telephone, in person, etc.) and the time spent with people of the user's “local social circle” (i.e., family and friends taking part in NESTORE). The interface clearly states whether these values come from a monitoring device (e.g., from the WOT agent connected to the smart bracelet) or from self-reporting. Finally, the mobile app provides the possibility to accept and schedule in the calendar the activities of the pathways proposed by the DSS. The mobile application is a convenient way to invite people to join and share an activity. If the invited person is not yet part of the NESTORE ecosystem, she/he will receive an e-mail notification and the possibility to register to NESTORE through the Social Platform.

Concerning the Social Platform, its main role is to connect NESTORE's users with other users and stay in touch with friends and family members. This is done through classic tools such as a forum and blog accessible via a simple web interface. Posts are regularly added to provide additional tips and tricks related to the four domains and use of NESTORE itself. In addition, through the “Events” page, the social platform shows geolocalized information about nearby events and activities that may interest the users. These events may be added by partner associations or by the users themselves.

**Figure 10:**
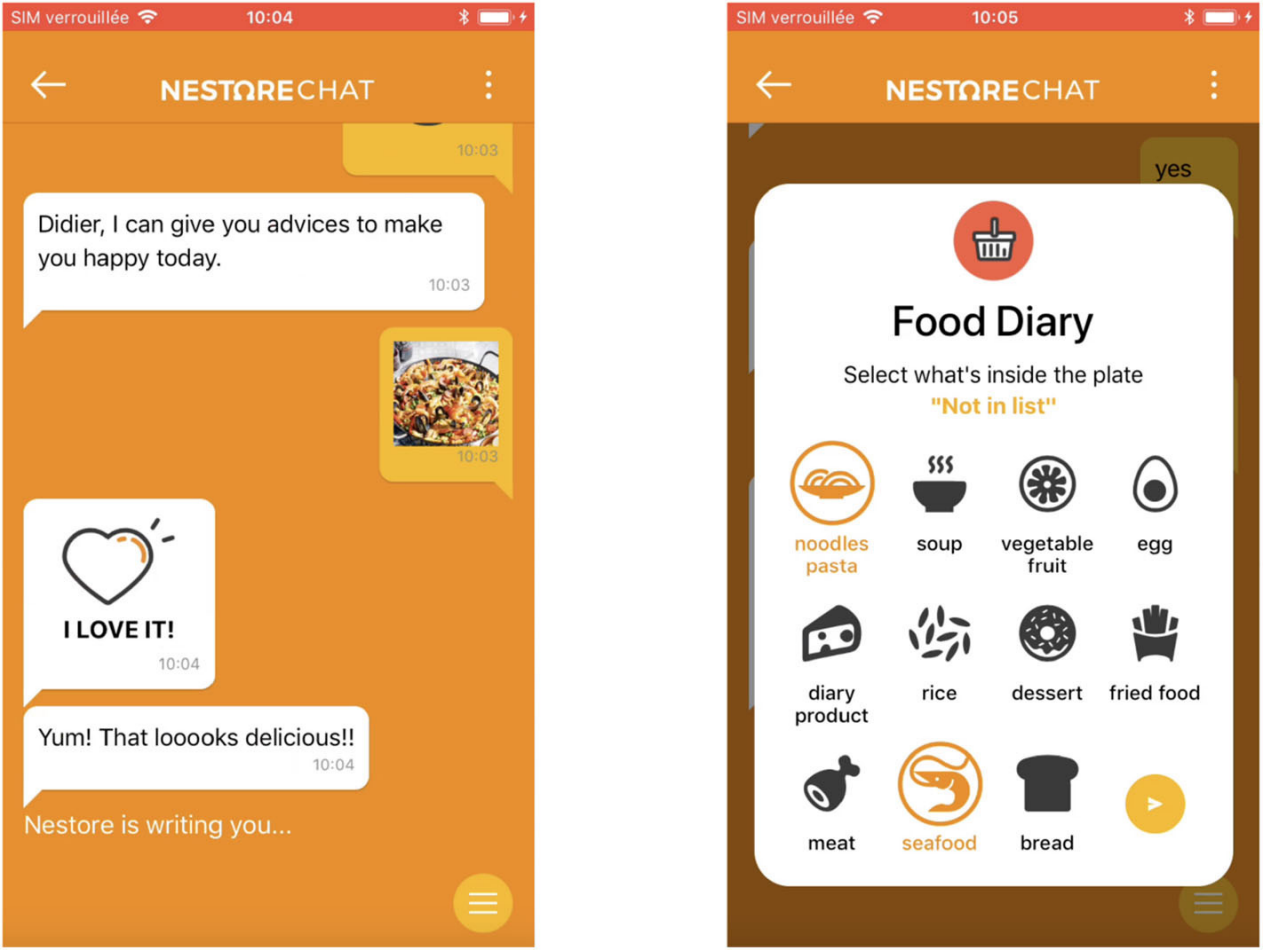
Meal logging via the chatbot interface.

Strictly interconnected with the mobile app, the chatbot provides an easy and intuitive interface to obtain personalized support (e.g., reminders, encouragement messages), receive coaching advice (e.g., instructions and tips), and enter personal information and preferences (e.g., during the registration process). The chatbot is also used to get a feedback from the user after an activity or to help him to comply self-reporting tasks. A notable example is the possibility to take pictures and log the current meal directly during a chat (see Figure 10). The questionnaires used to evaluate the user's status in the different domains are also administered via the chatbot. In this case, the interface changes according to the needs of the questionnaire (Likert-like scale, multiple choice answers, open questions, etc.) to provide the optimal response format.

In parallel with the app, a serious game has been developed to foster users' motivation (45). The serious game tackles specific subdomains such as strength, balance, and flexibility training for the physical domain or spatial navigation, inhibition and visuo-motor functioning for the cognitive domain (46). This is done thanks to an in-game virtual character (called Nestor) leading the user through a series of mini-games and guided exercises. Serious game sessions are suggested by the DSS and added to the user's calendar similarly to any other coaching activity.

**Figure 11:**
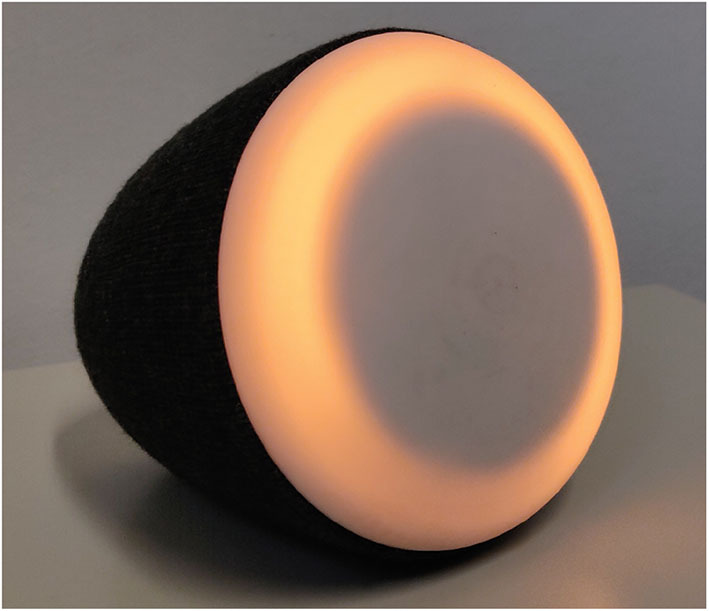
The tangible interface.

The tangible interface is connected to the same modules that manage the chatbot. This allows implementing a conversational agent with the same characteristics and behavior of the chatbot but based on vocal interaction. This solution allows to have a coherence in terms of a user's perception of how the e-coach behaves and interacts with his/her despite being embodied in different devices and using different modalities. The tangible interface is a physical object designed for domestic use (Figure 11); it has a particular shape that allows for placement in two different positions: one vertical with the wide base down, and one horizontal with the wide base toward the user. The first position, the vertical one, corresponds to the sleep position, which implies that the e-coach will not disturb the user. The second position (horizontal) means that the user is open to interact with the e-coach. The wide base integrates LEDs that are controlled by the system in order to provide different patterns that accompany different behaviors of the e-coach and to display different types of information. Therefore, the horizontal position has been designed considering that the user can see the LEDs on the base in order to receive complementary information. The behavior of the tangible interface intends to be life-like in order to establish an affective relationship with the user for a long-lasting bonding. The enclosure is covered with a soft textile to provide multisensorial experience while interacting with it, and the textile provides a sensation of warmth that the user can associate to the embodiment of the e-coach in a domestic environment, which should be intimate, cozy, and relaxing. The interaction with the tangible interface wants to replicate these sensations of intimate, cozy, and relaxing experience. The e-coach is wireless and has a base to charge its battery. In order to be positioned on the charging station, the tangible interface has to be in vertical position, which implies that it is in sleep mode. The vocal interface is not only convenient for the interaction while performing other tasks, but it also provides the opportunity for sentiment analysis, which is used to understand affective processes in order to adapt the conversation flow and the behavior of the tangible interface (e.g., acknowledgment + encouragement).

## 5. Validating the NESTORE System

The validation of the NESTORE system has to address the question of whether the proposed and developed system will indeed serve the intended purpose of having a positive impact on well-being and health, and show that the different components and tools are safe and practical in their use. Likewise, validation must offer information on the feasibility of carrying out a clinical trial to demonstrate the effectiveness of the system in achieving its objective of promoting healthy aging, quality of life and well-being.

The study design was selected considering that the user needs to adopt the NESTORE system for at least 3 months to allow a sufficiently long time period for the interventions to elicit changes in the respective coaching domains. Moreover, in order to assure that any observed effects can be attributed to the NESTORE intervention specifically, and not just to the fact that individuals have engaged in anything new, a well-defined active control group is necessary that engages in activities comparable with the NESTORE system, but without the “active ingredients” of the NESTORE coach (i.e., personalization and technology-based features). One feasible solution that will be followed in the initial pilot study is to deliver printed general recommendations about healthy habits in all NESTORE domains (nutrition/physical activity/cognition/social) to the control group, which would allow a comparison between the personalized and technology-based NESTORE interventions with more traditional general (i.e., non-personalized) and paper-based coaching material.

**Figure 12:**
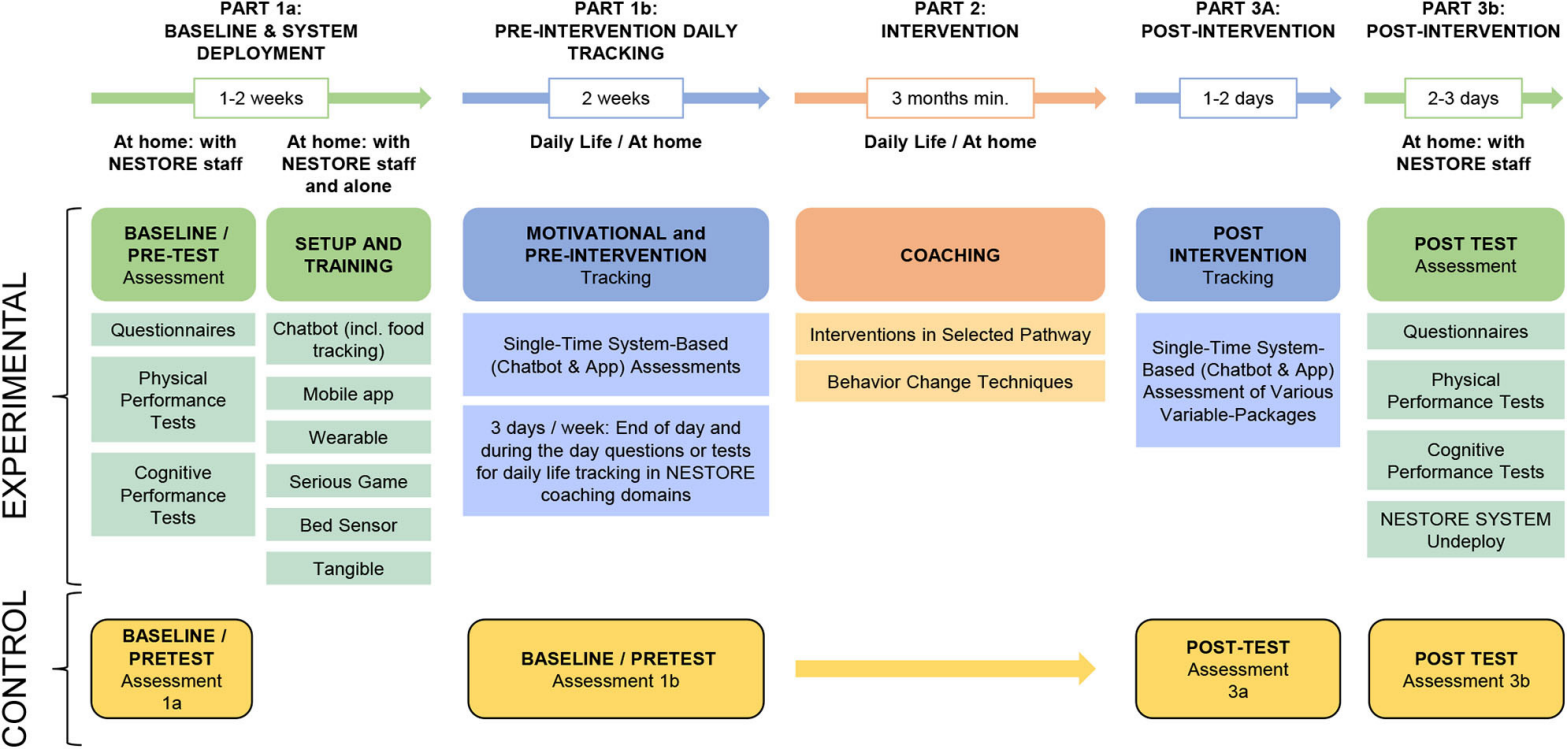
NESTORE Study Design: Experimental and Control Group.

All objectives will be addressed by the design outlined in Figure 12 that describes the data collection flow carried out in the three NESTORE pilot sites (Spain, The Netherlands, and Italy). We will use a pre-/post-test study comparing the group of individuals that receives the technology-based and personalized health coaching intervention to a control group that receives a standard, non-personalized intervention. The pilot-study will consist of the following phases (1) pre-test assessment, (2) setup of & training with system, (3) motivational and pre-intervention tracking phase in daily life, (4) coaching phase, (5) post-intervention tracking, and (6) post-test assessment.

The overall sample consists of a convenience sample of 60 persons (20 per site) using the NESTORE system and 30 controls (10 per site), gender balanced (15 men and 15 women at each site). NESTORE users need to be cognitively, physically and mentally healthy enough to be able to use the system. At the same time, the system is developed for healthy older adults who do not have certain conditions that could possibly make the interventions harmful to themselves. Thus, all chronic diseases (cardiovascular, metabolic, neurological, immunologic) are an exclusion criterion. Only persons with certain controlled chronic conditions can be included as they could benefit from the NESTORE system or the NESTORE system will suggest prospective users to turn to their primary physician to support in managing their conditions. Participants will be recruited with age between 65 and 75 years, living alone or community dwelling in autonomy, with ability to use standard ICT devices and availability of internet connection at home.

### 5.1. Preliminary Findings

The fieldwork started on February 2020 with the baseline assessments and the first installations of the intervention group. A prototype of the Personalized Coach kit has been deployed in two Pilot sites of the NESTORE project in Barcelona (Spain) and Monza (Italy). Ten older people (4 Women, 6 Men) have been equipped with the kit before COVID-19 lock-down. After completing a maximum of 4 weeks of system use, 2 or 3 weeks of daily tracking and one or two of intervention, some lessons learnt emerged from this short period.

The preliminary results revealed two main aspects: the significance of a detailed and well-specified procedure is essential for the correct progress of the installation process and secondly, the significant failure of a part of the system affects the overall perception of the system, despite its good functioning.

Regarding the first finding, through the definition of a procedure were established the main needs to be covered in each of the 3 stages: *pre-installation* (where it is structured and planned all the necessary aspects related to deployment and installation like the definition of workflows, training materials and resources), *installation* (when all the steps are performed, but also it is reinforced the user training in the system use) and *post-installation* (when the system is completely deployed and prepared for the participant autonomous use). In the pre-installation it was considered crucial to do a user-centered installation that means respectful with the daily rhythms, reducing the presence in their homes and based in personalize attention of their needs. During the installation, an ordered written procedure set the steps to avoid mistakes. Furthermore, it was video recorded the first installation as a model for the rest of installations. And finally, the post-installation was encompassed a non-intrusive method to monitor the data flow to assure that the system was working correctly.

Some qualitative results were obtained before the intermediate assessment thanks to the voluntarily self-report of users using the provided channels by researchers. From a performance point of view, the first results showed that it is critical the information that users received before installation. When users started to use the system, they modified their perception related to the system usefulness, because their expectancy did not match with the real life first impressions of use. However, it is important to note that the project was stopped just in the moment when participants were familiarizing and learning how to use the system.

In reference to the sensing layer the first findings demonstrated that the wristband is one of the system devices that centered more interest in users during the recruitment stage. The first results suggest a corpus of perceptions which created a framework providing significant opportunities to improve certain aspects in functional and non-functional areas described in the following:

- *Power consumption*: Although, the battery life is 18 h, the comparison with commercial solutions affect the participant' perception about the device reliability.
- *Battery charge base*: In the post-installation phase, some participants reported problems to the correctly place of the wristband in the charging base, even though it was provided a manual and they were instructed to do it.
- *Monitoring the battery status*: Uncertainty generated in some participants when the battery life is less than expected.
- *Switch system*: reported difficulties to turn on and off the wristband after the training session.

With respect to the software layer, some barriers were reported by users after the daily tracking described in the following:

- *Exceeding the push notifications number over user's expectations*: The theoretical number of push notifications is not in accordance with the real users' tolerance frame. But also, the findings showed that a group of participants were unconscious of receiving them, and a reduce part of participants informed that they deliberately ignored them after few days. On the other hand, users did not appreciate receiving duplicate notifications and affected their satisfaction perception.
- *Connection between NESTORE coach app and NESTORE connect app*: When the participant is prepared to perform some formal tests using the NESTORE app, the communication between it and the NESTORE connect create a loop that generate the perception that something is not working well. It happened very often in the beginning of the pilot study because some Android functionalities in the firmware.
- *Task repetition and workload during intervention*: Users reported the feeling that they always have done the same type of task related to the intervention like fulfill questionnaires, but also some tasks were reported as heavy.
- *Motivation triggers*: The system did not integrate enough mechanisms a part of notifications that could sustain over the intervention the users' interest to complete new activities. During the co-design participants asked for having a model of coaching friendly, but the prototype is not perceived in this direction by the moment.
- *Usefulness perception of NESTORE Coach app*: Some functionalities were not used during the first weeks of the intervention because there were some technical incidences. On the other hand, participants reported problems using the nutrition app or the cognitive tests. These problems but also the unbalance between the time invested to do tasks and the benefit directly obtained from the system used, affected they usefulness perception after the daily tracking period.

## 6. Discussion and Conclusions

The NESTORE virtual coach personalized strategies includes self-monitoring, context-awareness, and different interface modalities to better address when, how, and what coaching messages to deliver to the user in an automated and intelligent way. However, besides the significant challenges related to the optimization and orchestration of the different hardware (i.e., set of devices) and software (i.e., back-end modules) artifacts involved in the system, it is important to provide to the user a valid tool, as a virtual companion, in terms of usability and effectiveness. For these reasons, we presented the overall architecture of the NESTORE system, the different hardware and software modules enabling the virtual coaching, and a validation study about the feasibility of carrying out a clinical trial to demonstrate the effectiveness of the system in achieving its objective in promoting healthy aging, quality of life, and well-being.

Since the design phases of the system, we put the muldi-domain approach at the core of NESTORE both in the modeling of the end-user and in the development of the different modules in the back-end. Each service, thanks to the modularity offered by the IoT architecture, exchanges information about related domain which is then orchestrated by the DSS to provide to the user personalized pathways of well-being. This approach has its roots in the participatory design techniques adopted, in terms of co-design and co-creation of the offered services. In this vision, we also embraced the technological outcomes from the community created around EU programme on “Active aging and self-management of health,” offering an IoT architecture supporting the integration with other strategic projects addressing similar objectives.

## Data Availability Statement

The original contributions presented in the study are included in the article/supplementary material, further inquiries can be directed to the corresponding author/s.

## Author Contributions

FP developed the idea and led this writing of the manuscript. AC, FF, and MG contributed to the section related to the integrated infrastructure. AM, GM, CR, SG, AS, and GR described the personalized coaching experience as domain experts. MC, FC, OA, and EM contributed to the description of the virtual coach. EM, ED, MM, and DW contributed to the description of the monitoring system. PS-B and SO described the decision support system. CC and GC contributed to the description of the IoT architecture. All authors edited the drafts of the manuscript and provided critical revisions.

## Conflict of Interest

ED, MM, and DW were employed by company FLEXTRONICS DESIGN SRL, while CC and GC were employed by company ROPARDO SRL. The remaining authors declare that the research was conducted in the absence of any commercial or financial relationships that could be construed as a potential conflict of interest.

## Footnotes

^1^H2020 call: H2020-SC1-2016-2017, topic SC1-PM-15-2017, partners from: Belgium, Italy, Netherlands, Romania, Spain, Switzerland, United Kingdom.

^2^ http://www.activeageing.unito.it/en/home

^3^https://cordis.europa.eu/project/rcn/194088/es

^4^http://radio-project.eu/

^5^ http://www.preventit.eu/

^6^http://reach2020.eu/

^7^https://grafana.com/
